# Increased Rates of Vitamin D Insufficiency in Boys With Duchenne Muscular Dystrophy Despite Higher Vitamin D_3_ Supplementation

**DOI:** 10.1177/2333794X19835661

**Published:** 2019-03-15

**Authors:** Qian Bian, Laura McAdam, Marc Grynpas, Jane Mitchell, Jennifer Harrington

**Affiliations:** 1 Department of Pharmacology and Toxicology, University of Toronto, Toronto, Ontario, Canada; 2 Holland Bloorview Kids Rehabilitation Hospital, Toronto, Ontario, Canada; 3 Lunenfeld-Tanenbaum Research Institute, University of Toronto, Toronto, Ontario, Canada; 4 The Hospital for Sick Children, University of Toronto, Toronto, Ontario, Canada

**Keywords:** duchenne muscular dystrophy, vitamin D, fractures, osteoporosis

## Abstract

Vitamin D supplementation is important for many chronic pediatric conditions to help maintain bone health; however, there is little evidence about how disease-related factors affect vitamin D status. The objective was to compare 25-hydroxyvitamin D (25(OH)D) concentrations in 3 pediatric cohorts (Duchenne muscular dystrophy [DMD], systemic lupus erythematosus [SLE], and osteogenesis imperfecta [OI]). In a retrospective study of 367 subjects, children with DMD had increased prevalence of vitamin D insufficiency (25% vs 14% [SLE] and 10% [OI], *P* = .002), despite higher vitamin D_3_ supplementation doses. Boys with DMD also had higher weight, fat mass, and lower lean mass percentage *Z* scores. DMD was associated with having higher rates of vitamin D insufficiency than other comparable pediatric chronic disease cohorts, the effect of which may be modulated by clinical factors such as increased adiposity. While corroboration of these results is needed given baseline differences between the patient groups, greater vitamin D supplementation doses may be required to achieve optimal serum 25(OH)D concentrations in boys with DMD.

## Introduction

Bone fragility is a recognized complication of many pediatric chronic conditions.^1^ Consequences of bone fragility, including vertebral and long bone fractures, frequently lead to significant morbidity including pain and decreased ambulation.^2-4^ Maintenance of vitamin D sufficiency, as measured by the major circulating metabolite 25-hydroxyvitamin D (25(OH)D), is important to facilitate bone mineralization and maintain bone health. While there are recommendations about vitamin D supplementation in healthy children,^5-7^ information about dosing needed to maintain vitamin D sufficiency in differing pediatric chronic disease cohorts is limited.

Duchenne muscular dystrophy (DMD), an X-linked recessive condition caused by mutations in the dystrophin gene, is associated with progressive skeletal and cardiac muscle weakness.^8,9^ Chronic glucocorticoid therapy has been demonstrated to prolong ambulation, preserve cardiac and pulmonary function, and significantly reduce the rate of scoliosis.^10-12^ Boys with DMD have a significant increased risk for bone fragility and fractures. Studies have demonstrated that low-trauma long bone fractures occur in 20% to 40% and symptomatic vertebral fractures in 15% to 30% of children with DMD.^13-15^ When children are screened for asymptomatic vertebral fractures, the rate of vertebral height loss in this patient population is even higher, up to 53%.^16^

Previous studies have reported rates of vitamin D deficiency of 35% in DMD patients despite supplementation.^13,17^ While poor adherence with vitamin supplementation is a frequent issue in children with chronic diseases, glucocorticoid treatment has also been associated with significantly lower serum 25(OH)D concentrations. In a mouse model of DMD, glucocorticoid treatment was associated with a 40% decrease in serum 25(OH)D concentrations.^18^ Increased adiposity, a frequent finding in boys with DMD, has also been shown to be inversely associated with serum 25(OH)D concentrations.^19^ Further information is needed to understand the individual as well as disease characteristics contributing to this high rate of vitamin D insufficiency, and how this rate compares with other children with similar chronic conditions. Assessing the relationship between vitamin D status to disease and treatment characteristics would therefore help inform recommendations regarding vitamin D supplementation in children with chronic conditions.

In order to look at potential individual and disease determinants of serum 25(OH)D concentrations in different pediatric chronic disease cohorts, we aimed to compare the vitamin D status in boys with DMD with 2 other patient cohorts; patients with systemic lupus erythematosus (SLE) who also are frequently treated with chronic glucocorticoids; and second, patients with osteogenesis imperfecta (OI) who have a similar degree of counseling around the importance of vitamin D for bone health, and a subset of whom have decreased ambulation, similar to boys with DMD. All 3 cohorts of children are prescribed vitamin D_3_ supplementation as part of their routine medical care. The objectives of this study were to assess the 25(OH)D concentrations in a cohort of boys with DMD compared with children with SLE and OI, and to determine whether anthropometric, ambulatory, or disease-related factors influence vitamin D status.

## Methods

This was a retrospective study of children with DMD followed at Holland-Bloorview Kids Rehabilitation Hospital and children with SLE and OI followed at The Hospital for Sick Children, Toronto, Canada. Medical charts were reviewed for children who had been seen between January 1, 2008, and December 31, 2014. Patients from the DMD population had their disease diagnosis confirmed via gene analysis or muscle biopsy, children with SLE if they fulfilled at least 4/11 criteria of the American College of Rheumatology classification for SLE,^20^ and a diagnosis of OI was based on either the presence of a known causative OI genetic mutation or a clinical diagnosis in a child with classical phenotypical and radiological features. To be included in the study, each patient had to have a minimum of 1 serum 25(OH)D measurement. When multiple measurements were present, the biochemistry data point closest to December 31, 2014, was included as the sole 25(OH)D measurement for this study. In total, 83 children with DMD, 90 with OI, and 194 with SLE were included in the analysis.

### Clinical Data

Information was collected from the patients’ records at the time point closest to the 25(OH)D measurement. Data collected included age, sex, as well as variables related to the underlying condition (age of diagnosis, duration of disease), and treatment factors (duration and dose of glucocorticoids, other medications). Vitamin D_3_ supplementation dose that had been prescribed 6 months prior to the 25(OH)D sample was recorded. Given OI and DMD are genetic conditions, disease duration was calculated from birth, while for the children with SLE, duration was calculated from date of diagnosis. Boys with DMD were treated with the glucocorticoid deflazacort following the current Canadian standards.^21^ To compare the glucocorticoid exposure between the DMD and SLE cohorts, all glucocorticoid doses were converted to mg/kg of prednisone equivalents. Ambulatory status was evaluated in children with DMD using the Vignos scale, a qualitative scale where a value of “1” represents complete independent ambulation, and “9” representing wheelchair bound.^22^ In the children with OI and SLE, ambulatory status was categorized as either independent, able to ambulate with assistance (eg, using walkers), or wheelchair dependent. In order to compare the 3 groups, Vignos scores 1 to 4 were grouped into the independent ambulation group, and 5 to 8 into ambulate with assistance. Height, weight, and body mass index (weight [kg]/height^2^ [m]) *z* scores were calculated using reference data provided by the World Health Organization standardized reference charts. To account for potential seasonal variation in 25(OH)D concentrations, the month the sample was collected was recorded and categorized as either summer (June to August), fall (September to November), winter (December to February), or spring (March to May).

Lumbar spine (L1 to L4) and total body (not including head) bone mineral density (BMD) were measured by dual-energy X-ray absorptiometry (DXA) in the anterior-posterior direction (Lunar Prodigy; General Electric, Madison, WI). All measured values were transformed into *Z* scores using the equipment-specific age- and sex-adjusted Canadian reference values. Height *Z* score was used to calculate height-adjusted BMD *Z* scores, as previously described.^23^ Whole body DXA scans were also used to calculate body composition measures: body fat and lean mass percentage. Fat mass *Z* scores as well as lean mass corrected for height (lean mass/height^2^) *Z* scores were calculated using reference values from the National Health and Nutrition Examination Survey.^24^

### Statistical Analysis

The primary outcome measures were serum 25(OH)D concentration and serum 25(OH)D per IU of vitamin D_3_ supplementation. Differences in 25(OH)D concentrations and baseline characteristics between the 3 cohorts were compared using 1-way ANOVA (analysis of variance) followed by Bonferroni post hoc analysis or χ^2^ test. Correlations between clinical variables with the primary outcome measures were evaluated with Pearson and Spearman rank correlations. Simple linear regression analysis was used to identify predictors of serum 25(OH)D and serum 25(OH)D per IU of vitamin D supplementation. The data from all 3 cohorts were combined into one unified cohort, and multiple linear regression analysis was performed to determine biological and pharmacological determinants of serum 25(OH)D and serum 25(OH)D per IU of vitamin D_3_ supplementation. Predictor variables with a *P* value of less than .1 in the univariate test were included in the multiple linear regression. Statistical significance was inferred with a *P* value less than .05. The data were analyzed using SPSS 24.0 for windows (SPSS Inc, Chicago, IL).

### Ethical Approval and Informed Consent

This study was approved by the Hospital for Sick Children’s (REB# 1000049214) and Holland-Bloorview Kids Rehabilitation Hospital’s (REB# 15-619) Research Ethics Committees. Approval to waive the need for informed consent was given by the Ethics Committees, given the retrospective nature of the chart review.

## Results

The clinical characteristics of the 3 patient cohorts are summarized in Table 1. Eighty-seven percent of the boys with DMD and 75% of the SLE cohort were taking glucocorticoids at the time of the data collection. In the cohort of children with OI, 46% had a mild phenotype, 25% moderate, and 29% significant deforming OI, and 82% of the children had received bisphosphonate therapy. While calcium concentrations differed between the 3 groups, all values were in the normal range.

**Table 1.** Cohort Characteristics of the 3 Patient Groups*.

	DMD (n = 83)	OI (n = 90)	SLE (n = 194)	ANOVA *P*
Cohort characteristics				
Age (years)	10.3 (3.8)	8.7 (4.8)	15.6 (2.6)	<.001^a^
% Males	100	50	22	<.05^b^
Season of visit (% summer: fall: winter: spring)	37:16:25:22	21:41:18:20	29:22:26:23	NS
Ambulatory status (% independent: with assistance: wheelchair dependent)	68: 16: 16	74: 13: 13	100: 0: 0	<.05^b^
Height (*Z* score)	−1.7 (1.8)	−1.5 (2.0)	−0.4 (1.3)	<.001^a^
Weight (*Z* score)	−0.6 (1.5)	−0.5 (1.5)	0.3 (1.3)	<.001^a^
BMI (*Z* score)	0.4 (1.6)	0.5 (1.4)	0.7 (1.2)	NS
Glucocorticoid dose (mg/kg/day of prednisone equivalent)	0.48 (0.27)	0	0.13 (0.18)	<.001^a,c^
Serum biochemistry				
Total calcium (mmol/L)	2.40 (0.1)	2.51 (0.1)	2.38 (0.1)	<.001^c^
Phosphate (mmol/L)	1.53 (0.2)	1.58 (0.2)	1.36 (0.2)	<.001^a^
DXA values				
Lumbar spine BMD (*Z* score)	−2.6 (1.3)	−0.9 (1.4)	−0.8 (1.2)	<.001^a,c^
Height-adjusted lumbar spine BMD (*Z* score)	−1.6 (1.4)	−0.2 (1.5)	−0.5 (1.1)	<.001^a,c^
Total body BMD (*Z* score)	−4.3 (2.0)	−1.0 (1.4)	−0.4 (1.2)	<.001^a,c^
Height-adjusted total body BMD (*Z* score)	−2.8 (2.5)	−0.1 (1.5)	0.0 (1.0)	<.001^a,c^
Body fat (% of total weight)	39.1 (12.9)	28.3 (10.8)	33.4 (10.4)	<.05^a,c^
Body fat % (*Z* score)	1.0 (1.3)	−0.5 (2.0)	0.2 (1.5)	<.001^a,c^
Lean mass (% of total weight)	59.3 (12.6)	68.2 (10.1)	62.8 (110.2)	<.001^c^
Lean mass/height^2^ (*Z* score)	−3.1 (2.1)	−0.6 (1.2)	−0.7 (1.1)	<.001^a,c^

Abbreviations: DMD, Duchenne muscular dystrophy; OI, osteogenesis imperfecta; SLE, systemic lupus erythematosus; ANOVA, analysis of variance; NS, not significant; BMI, body mass index; DXA, dual-energy X-ray absorptiometry; BMD, bone mineral density.

* Results are recorded as mean (SD).

Statistical significance across the 3 groups analyzed using ANOVA with Bonferroni post hoc analysis where ^a^*P* < .05 when comparing DMD to SLE; ^b^statistical significance across the 3 groups analyzed groups using χ^2^ tests; ^c^*P* < .05 when comparing DMD to OI.

There were significant differences in body composition across the 3 cohorts. Although body mass index *Z* score did not differ, the boys with DMD had a greater weight and body fat percentage with lower lean mass/height^2^ *Z* scores. Body composition differed by ambulatory status, with higher body fat and lower lean mass *Z* scores in the nonambulatory children (*P* < .001). Higher glucocorticoid dose was also associated with higher fat percentage *Z* score (*r* = 0.35, *P* < .001).

The boys with DMD had significantly lower lumbar spine and total body BMD *Z* scores, including when adjusted for height *Z* score, compared with children with OI and SLE (Table 1). In the DMD cohort, 34% and 63% had a BMD *Z* score of less than −2 of the lumbar spine and total body, respectively. Both lumbar spine and total body BMD *Z* scores decreased with age (*r* = −0.46, *P* < .0001; and *r* = −0.32, *P* = .02, respectively); however, when BMD was corrected for height *Z* score, this relationship did not persist.

To ensure that sex was not a significant contributor to the differences seen, the same variables in Table 1 were compared between the boys with DMD, to the boys with OI (n = 46) and SLE (n = 42). The significant differences across the 3 cohorts as well as the intergroup post hoc analyses remained the same.

### Comparison of Vitamin D Status of DMD, SLE, and OI Populations

**Figure 1.**
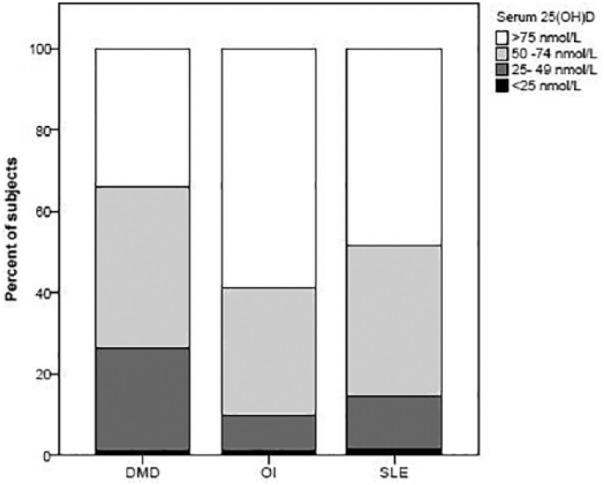
Distribution of vitamin D concentrations across the 3 cohorts. A significantly greater percentage of the boys with DMD (Duchenne muscular dystrophy) had 25-hydroxyvitamin D levels less than 50 nmol/L compared with children with OI (osteogenesis imperfecta) or SLE (systemic lupus erythematosus).

A significantly greater percentage of the boys with DMD had 25(OH)D levels less than 50 nmol/L compared with the other patient populations (25% vs 10% in OI and 14% in SLE, *P* = .002; Figure 1). The mean 25(OH)D concentration in the DMD cohort was also significantly lower, despite being prescribed higher vitamin D_3_ supplementation doses (Table 2). As a result, the 25(OH)D concentration corrected for vitamin D supplementation dose was also significantly lower in the boys with DMD. These findings were the same when the analysis was limited to data from the males in the OI and SLE groups (Table 2).

**Table 2.** Comparison of 25(OH)D Concentrations and Vitamin D Supplementation Across the 3 Patient Groups*.

Vitamin D Characteristics	DMD (n = 83)	OI (n = 90)	SLE (n = 194)	ANOVA *P*
25(OH)D (nmol/L)	65.1 (21.4)	82.6 (29.2)^a^	76.8 (26.6)^b^	<.001
Males only	65.1 (21.4)	86.5 (30.8)^a^	78.5 (28.0)^b^	
Vitamin D supplementation (IU)	1627 (1011)	698 (441)^a^	1006 (339)^b^	<.001
Males only	1627 (1011)	780 (471)^a^	978 (270)^b^	
Vitamin D supplementation per weight (IU/kg/day)	63.3 (53.0)	29.1 (23.9)^a^	18.9 (9.0)^b^	<.001
Males only	63.3 (53.0)	28.0 (17.6)^a^	16.0 (6.1)^b^	
25(OH)D per IU of vitamin D_3_ supplementation (nmol/L/IU)	0.05 (0.02)	0.15 (0.1)^a^	0.09 (0.07)^b^	<.001
Males only	0.05 (0.02)	0.15 (0.1)^a^	0.10 (0.07)^b^	

Abbreviations: 25(OH)D, 25-hydroxyvitamin D; DMD, Duchenne muscular dystrophy; OI, osteogenesis imperfecta; SLE, systemic lupus erythematosus; ANOVA, analysis of variance.

* Results are shown in mean (SD). Comparison of variables utilizing data from males only in the OI (n = 46) and SLE (n = 42) cohorts is also shown. 1 IU of vitamin D_3_ = 0.025 µg.

a *P* < .05 when comparing DMD to OI.

b *P* < .05 when comparing DMD to SLE using Bonferroni post hoc analysis.

### Clinical Correlates of Vitamin D Status

**Figure 2.**
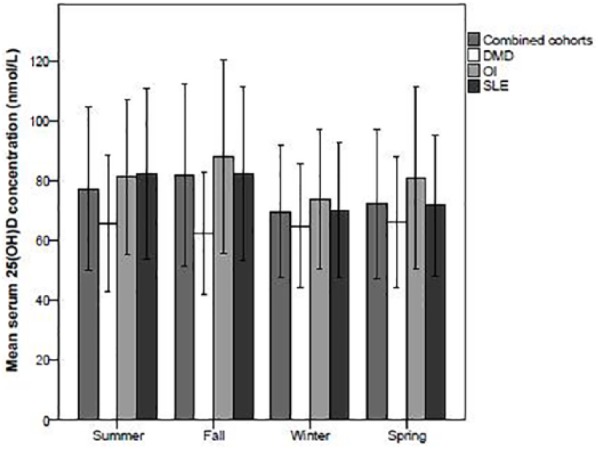
Seasonal variation in 25-hydroxyvitamin D (25(OH)D) concentrations. In the combined 3 cohorts, there was a seasonal variation in serum 25(OH)D concentrations, with the lowest concentrations measured in winter. Comparing the separate groups, the difference in 25(OH)D concentrations by season was, however, only seen in the patients with SLE (systemic lupus erythematosus), and not in the OI (osteogenesis imperfecta) or DMD (Duchenne muscular dystrophy) cohorts. The error bars indicate ±1 standard deviation.

Combining the 3 cohorts, there was a seasonal variation in serum 25(OH)D concentrations, with the lowest concentrations measured in winter (*P* = .01, Figure 2). Comparing the separate groups, the difference in 25(OH)D concentrations by season was, however, only seen in the patients with SLE (*P* < .001) and not in the OI or DMD cohorts. In the boys with DMD, higher vitamin D_3_ supplementation was associated with increased serum 25(OH)D (*r* = 0.28, *P* = .01). This association between vitamin D supplementation and serum 25(OH)D levels was not seen in the children with OI or SLE, potentially indicating that serum 25(OH)D concentrations are influenced by other sources of vitamin D in these patient groups. Analyzing the combined study population, the clinical correlates with serum 25(OH)D concentration as well as 25(OH)D per IU of vitamin D_3_ supplementation are displayed in Table 3. Multiple linear regression demonstrated that the significant predictors for serum 25(OH)D and 25(OH)D per IU of vitamin D_3_ supplementation included a diagnosis of DMD (β = −12.2 [range = −19.6 to −4.8]), disease duration (β = −0.9 [range = −1.6 to −0.2]), weight *Z* score (β = −2.8 [range = −4.8 to −0.8]), and having a 25(OH)D collected in the fall (β = 8.6 [range = 2.0 to 15.2]).

**Table 3.** Clinical Correlates in the Combined Cohort With the Primary Outcomes.

Clinical Variable	Serum 25(OH)D (nmol/L)		Serum 25(OH)D per IU of Vitamin D_3_ Supplementation	
	*r*	*P*	*r*	*P*
Age (years)	−0.10	.05	−0.16	.003
Disease duration (years)	−0.19	<.001	−0.13	.02
Glucocorticoid dose (mg/kg)^a^	−0.05	.44	−0.17	.007
Weight (*Z* score)	−0.09	.09	−0.10	.07
BMI (*Z* score)	0.03	.64	−0.05	.36
Fat mass % (*Z* score)	−0.06	.36	−0.16	.02
Lean mass/height^2^ (*Z* score)	−0.06	.36	0.15	.03
Total calcium (mmol/L)	0.09	.08	0.18	.001

Abbreviations: 25(OH)D, 25-hydroxyvitamin D; BMI, body mass index; DMD, Duchenne muscular dystrophy; SLE, systemic lupus erythematosus; OI, osteogenesis imperfecta.

a Correlation done only using DMD and SLE cohorts as no child with OI on glucocorticoid treatment.

## Discussion

Maintenance of vitamin D sufficiency in children at increased risk for bone fragility is an important modifiable risk factor. Little data exist about disease and treatment characteristics that may affect vitamin D status between chronic disease cohorts. Our data demonstrate that boys with DMD have higher rates of vitamin D insufficiency compared with children with OI and SLE, despite significantly larger vitamin D_3_ supplementation doses. Twenty-five percent of the children with DMD had serum 25(OH)D concentrations less than 50 nmol/L, a prevalence that is comparable to previous studies, which have reported rates of vitamin D insufficiency of around 35%.^13,17^ In contrast to these studies, the mean vitamin D supplementation dose in our cohort was higher (approximately 1600 IU/day) compared with average supplementation doses in other studies of 1000 to 1200 IU/day. Thus, even on higher than standardly used vitamin D supplementation doses, there is a significant rate of vitamin D deficiency in boys with DMD.

The significant contributor to serum 25(OH)D concentrations in the DMD cohort was vitamin D_3_ supplementation dose, indicating that this is likely their primary source. While UV exposure was not directly measured, there was no seasonal variation of serum 25(OH)D in the boys with DMD potentially due to decreased outdoor time and sun exposure, as previously seen in other cohorts.^17^ A prospective trial by Bianchi et al of 33 boys with DMD supplemented with vitamin D for 2 years with 0.8 µg/kg/day (equivalent to approximately 32 IU/kg/day) resulted in a mean 25(OH)D concentration at the end of the trial of approximately 100 nmol/L.^25^ This study reported high adherence rates to supplementation of 82%. Given that our patients with DMD were on average receiving double the vitamin D dose per kg, there must be other factors that affect why in a real-life setting, outside of a clinical trial, higher vitamin D supplementation doses are needed.

We had postulated that glucocorticoids may affect vitamin D status. While there was a linear association between glucocorticoid dose and serum 25(OH)D corrected for vitamin D dose, on multi-regression linear analysis, the association between glucocorticoids and serum 25(OH)D concentration did not persist. Increased weight *Z* score, however, was an important determinant of serum 25(OH)D concentrations, including when corrected for supplementation dose. Obesity has been identified as a significant issue in patients with DMD.^26^ Glucocorticoid treatment has been attributed as an exacerbating factor to this increased adiposity.^10^ Ambulatory status also appears contributing to this adiposity, given the higher body fat percentage *Z* score in the nonambulatory boys with DMD. Increased adiposity is associated with decreased bioavailability of vitamin D,^27,28^ and thus may be an important reason why boys with DMD need significantly higher vitamin D supplementation.

There are significant limitations to this study that warrant recognition. There was no quantification of dietary intake of vitamin D or sunlight exposure in our cohort, nor compliance with vitamin D supplementation. While vitamin D supplementation was standardly prescribed among the 3 disease cohorts, the level of perceived importance of taking it may have differed between the treating physicians and the families. Longitudinal assessment of vitamin D status and its association with changes in clinical variables may have revealed other important influencing factors. Other significant limitations of this study relate to the baseline differences between the 3 patient groups. While the group of patients with SLE were chosen as a glucocorticoid comparison patient population and because they were routine, there were significant differences in age, sex, and the dose of glucocorticoids. In addition, while the boys with DMD were typically on a constant steroid dose, the SLE subjects’ glucocorticoid dose often changed with disease activity. These inherent baseline differences between the groups may have affected the results, and these results therefore need further corroboration. In addition, given that inflammation may affect vitamin D metabolism, the amount of disease activity in the SLE population may also have influenced this groups’ results. A comparison of boys with DMD treated and not treated with glucocorticoids may have allowed better evaluation of the potential effect of glucocorticoids on serum 25(OH)D concentrations; however, given the small number of non–glucocorticoid-treated patients in this cohort, this was unable to be examined.

Bone fragility and fractures lead to significant morbidity in pediatric chronic disease cohorts including children with DMD. Vitamin D deficiency is a modifiable bone health risk factor, and optimal vitamin D status should achieved to help prevent demineralization of bone. Our data support the need for high vitamin D supplementation in boys with DMD, in order to maintain vitamin D sufficiency. Furthermore, clinical factors such as increased weight may have a role in explaining this increased need compared with other pediatric chronic disease cohorts.
